# Strongly Confined Spoof Surface Plasmon Polaritons Waveguiding Enabled by Planar Staggered Plasmonic Waveguides

**DOI:** 10.1038/srep38528

**Published:** 2016-12-05

**Authors:** Longfang Ye, Yifan Xiao, Yanhui Liu, Liang Zhang, Guoxiong Cai, Qing Huo Liu

**Affiliations:** 1 Institute of Electromagnetics and Acoustics, and Department of Electronic Science, Xiamen University, Xiamen 361005, China; 2 Shenzhen Research Institute of Xiamen University, Shenzhen 518057, China; 3 Department of Electrical and Computer Engineering, Duke University, Durham 27708, USA

## Abstract

We demonstrate a novel route to achieving highly efficient and strongly confined spoof surface plasmon polaritons (SPPs) waveguides at subwavelength scale enabled by planar staggered plasmonic waveguides (PSPWs). The structure of these new waveguides consists of an ultrathin metallic strip with periodic subwavelength staggered double groove arrays supported by a flexible dielectric substrate, leading to unique staggered EM coupling and waveguiding phenomenon. The spoof SPP propagation properties, including dispersion relations and near field distributions, are numerically investigated. Furthermore, broadband coplanar waveguide (CPW) to planar staggered plasmonic waveguide (PSPW) transitions are designed to achieve smooth momentum matching and highly efficient spoof SPP mode conversion. By applying these transitions, a CPW-PSPW-CPW structure is designed, fabricated and measured to verify the PSPW’s propagation performance at microwave frequencies. The investigation results show the proposed PSPWs have excellent performance of deep subwavelength spoof SPPs confinement, long propagation length and low bend loss, as well as great design flexibility to engineer the propagation properties by adjusting their geometry dimensions and material parameters. Our work opens up a new avenue for development of various advanced planar integrated plasmonic devices and circuits in microwave and terahertz regimes.

Surface plasmon polaritons (SPPs) are localized surface waves propagating along a metal-dielectric or metal-air interface at infrared or visible frequencies. Due to their ability to spatially confine electromagnetic waves in subwavelength scale, SPPs provide efficient solutions to circumvent the diffraction limit of conventional optical elements and build highly integrated optical components and circuits^1^,^2^,^3^. Microwave and terahertz radiations have great potential applications in communication, sensing, radar, spectroscopy and allow the analysis of various material properties^4^,^5^,^6^,^7^,^8^,^9^,^10^. Obviously, it may have many advantages if this concept of highly localized waveguiding can be applied to these lower frequency regimes (microwave or terahertz), where plasmonics enables high performance and deep miniaturization of waveguides, circuits and systems. In recent years, there has been an increasing interest into development of novel efficient plasmonic waveguides at lower frequencies. However, since the intrinsic plasma frequencies of most metals approach ultraviolet region, the natural SPPs along the flat and smooth metal-dielectric interface do not occur at lower frequencies, which severely restrict their practical applications in microwave or terahertz regimes.

In the last few years, in order to achieve strong subwavelength confinement waveguiding in microwave regime, the concept of spoof SPPs has been proposed to resemble the SPP behaviors at optical frequencies^11^. Based on this concept, various bulk plasmonic waveguides including perfect-conductor surfaces textured with subwavelength one-dimensional (rectangular, slanted, tapered, *etc.*) groove arrays^12^,^13^,^14^, or two-dimensional hole lattices^15^,^16^, metal wires with periodically corrugated ring arrays or helically groove arrays^17^,^18^,^19^,^20^ have been theoretically or experimentally demonstrated for their efficient and highly localized spoof SPP propagation at microwave or terahertz frequencies. Domino plasmonic waveguides, L-shaped, T-shaped, wedge-shaped or other lateral shaped plasmonic waveguides^21^,^22^,^23^,^24^,^25^ have also been investigated. The spoof SPP dispersion characteristics of these plasmonic waveguides can be directly manipulated by the shapes and dimensions of the subwavelength textures. Nevertheless, most of these structures have disadvantages of inherent bulk geometrics and some even require complicated manufacturing techniques in fabrication process. Recently, as a novel class of planar plasmonic waveguides for broadband and low loss spoof SPP propagation, periodic subwavelength corrugated ultrathin metallic strips have received extensive attention^26^,^27^,^28^,^29^. Compared with the bulk plasmonic waveguides, these corrugated ultrathin metallic strips have smaller planar structure and tighter field confinement and can be easily fabricated using the standard PCB process. Meanwhile, because of their good field confinement, the crosstalk from adjacent pairs of corrugated ultrathin waveguides is much lower than conventional microstrip lines^30^,^31^. Due to these attractive characteristics, corrugated ultrathin metallic strips provide a new way to achieve versatile planar plasmonic integrated circuits in lower frequency regime, especially at microwave or terahertz frequencies. To date, the properties of corrugated ultrathin metallic strips with single groove array or symmetrically double groove arrays have been theoretically and experimentally investigated, and based on them, many plasmonic devices including filters, splitters, antennas, and amplifiers have been demonstrated as well, which drastically accelerate the development of the spoof SPPs technology and their applications^26^,^27^,^28^,^29^,^30^,^31^,^32^,^33^,^34^,^35^,^36^,^37^,^38^,^39^. However, despite recent progress, the waveguiding properties of spoof SPPs propagating along ultrathin metallic strips with staggered double groove arrays still remain to be further investigated.

In this paper, planar staggered plasmonic waveguides (PSPWs) consisting of an ultrathin metallic strip with periodic subwavelength staggered double groove arrays supported by a flexible dielectric substrate are proposed as new promising plasmonic waveguides for efficient and strongly confined spoof SPP propagation at microwave and terahertz frequencies. By introducing such a staggered structure with a geometrically lateral shift, the proposed PSPWs demonstrate unique staggered EM coupling and spoof SPP waveguiding phenomenon. The spoof SPP propagation properties of these waveguides are numerically and experimentally investigated by taking into account the finite conductivity of the metal and the dielectric loss of the substrate. The near field distributions of spoof SPPs and dispersion relations versus geometry dimensions and material parameters are analyzed and compared. Further, broadband transitions with flaring ground and gradient depth grooves between CPW and the PSPW are designed to realize smooth momentum matching and highly efficient spoof SPP mode convention from CPW mode. By applying these transitions, a CPW-PSPW-CPW structure is designed, fabricated and measured to verify the PSPW’s propagation performance at microwave frequencies. The numerical and experimental results demonstrate the PSPWs have excellent performance with strong subwavelength confinement, long propagation length, and low bend loss for the spoof SPPs in a broad microwave frequency band from 1 GHz to 11.4 GHz. It is important to note that, due to the scale invariance of classical electromagnetism, the proposed PSPWs can be scaled up to the terahertz regime, which may have promising applications in integrated plasmonic devices in microwave and terahertz regimes.

## Results and Discussion

### Propagation properties of planar staggered plasmonic waveguides

**Figure 1:**
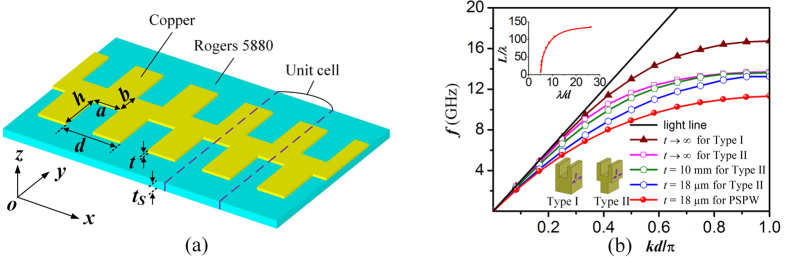
Schematic diagram and propagation characteristics of staggered plasmonic waveguide. (**a**) Schematic structure of PSPW consisting of a metallic strip with groove width *a* and depth *h* and period *d* supported by a flexible dielectric substrate. (**b**) Dispersion relations for the fundamental spoof SPP mode propagating along PSPW, single side corrugated copper plasmonic waveguide (inset: Type I) and staggered copper plasmonic waveguide (inset: Type II), and the top inset denotes the propagation length for PSPW, where the geometric parameters are fixed as *d* = 5 mm, *a* = 2.5 mm, *h* = 4 mm, *b* = 2 mm for all curves.

The proposed PSPW consists of an ultrathin metallic strip perforated by periodic subwavelength staggered double groove arrays on both sides printed on a flexible dielectric substrate. The schematic configuration of the PSPW is illustrated in Fig. 1(a), where the thickness of metallic strip is *t*, the separation and period of these two groove arrays of the metallic strip are *b* and *d*, and the width and depth of the grooves are *a* and *h*, while the relative permittivity and thickness of the dielectric substrate are *ε*_r_ and *t*_s_, respectively. To study the propagation characteristics along PSPWs in the *x*-direction, we use the Finite Element Method (FEM) to numerically calculate and analyze the dispersion relations and near field distributions under different geometric and dielectric parameters. Instead of assuming metallic strip as PEC and neglecting dielectric loss in many previous works, in this paper, copper with thickness of *t* = 18 μm and conductivity of 5.8 × 10^7^ S/m is selected as the metallic strip material, as well as Rogers RT/duroid 5880 with *t*_s_ = 0.787 mm, *ε*_r_ = 2.2 and loss tangent tan(δ) = 0.0001 is selected as the substrate material. As shown in Fig. 1(b), the dispersion curves of the fundamental mode for this proposed PSPW associated the frequency *f* with the propagation constant *k* are presented (see the red line with solid circle). Meanwhile, the dispersion curves corresponding to bulk copper plasmonic waveguide with a single-sided groove array (see inset in Fig. 1(b): Type I, *t* → ∞) and staggered copper plasmonic waveguides with different thicknesses (see inset in Fig. 1(b): Type II, *t* = 18 μm, 10 mm or *t* → ∞) without a dielectric substrate are also displayed for comparison. In these calculations, the geometric parameters are set as *d* = 5 mm, *a* = 2.5 mm, *h* = 4 mm, *b* = 2 mm for all curves. As observed in this figure, the asymptotic frequency of single side corrugated copper plasmonic waveguide (Type I) is 16.7 GHz when the thickness *t* is infinity. The asymptotic frequency of the staggered copper plasmonic waveguide (Type II) is much lower than the Type I waveguide, which decreases slightly from 13.7 GHz to 13.4 GHz as the *t* decreasing from ∞ to 18 μm. It is remarkable that the proposed PSPW consisting of both metallic strip and substrate exhibit even lower asymptotic frequency of 11.4 GHz than the plasmonic waveguides consisting of only metallic strip cases, indicating tighter field confinement. Clearly, all dispersion curves deviate far away from the light line, which is similar to the dispersion behaviors of SPPs in the optical regime. The top inset curve in Fig. 1(b) shows the corresponding propagation lengths of spoof SPPs propagating along this PSPW. It is found that the propagation length decreases as the operating frequency increases. This is because the fields of spoof SPPs are confined much tighter and causing more propagation loss in higher frequency band than in the lower one. Especially, when the operating frequency region is close to the asymptotic frequency, the propagation length is drastically decreased near to zero, resulting from the cut off phenomenon of the spoof SPP mode. Furthermore, to obtain the method for tuning spoof SPPs’ operating frequency bands, we further investigate the dependence of the dispersion relations on the geometric and dielectric parameters of PSPWs (see Supplementary Information). It is found that the dispersion curves will be dramatically tuned by varying the groove depth *h* and period *d* of metallic strip, as well as by changing the dielectric permittivity *ε*_r_ and thickness *t*_s_ of the substrate. The asymptotic frequency decreases rapidly as *h, d, ε*_r_ *and t*_s_ increase, demonstrating huge design flexibilities to engineer the spoof SPP dispersion relations. Therefore, by tuning the geometric parameters and dielectric parameters, the operating frequency band of PSPW can easily be extended to terahertz or infrared regimes.

**Figure 2:**
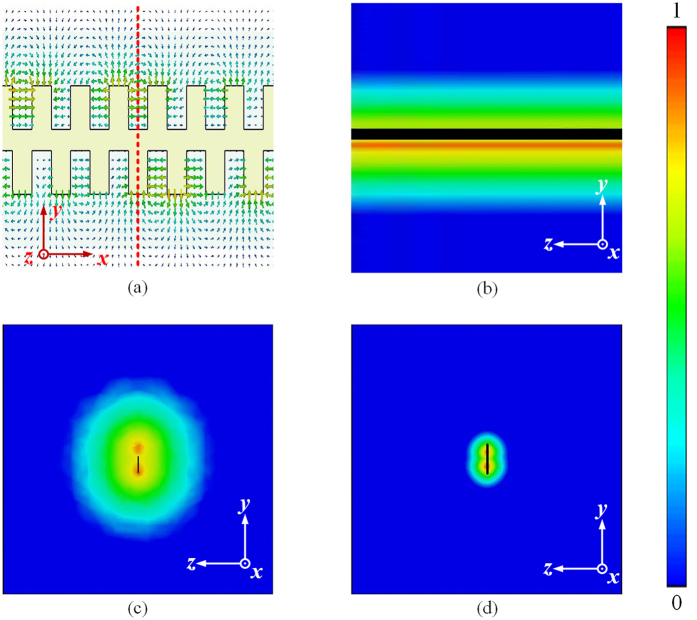
Simulated near field distributions for PSPW and staggered plasmonic waveguides (Type II). (**a**) Electric field vector distribution scheme at 11.4 GHz on the *xoy* plane cut in copper; (**b–d**) Normalized amplitudes of electric fields (|*E*|) distributions on the cross-section (*yoz* plane) for the staggered waveguide with *t* → ∞ without substrate (**b**) *t* = 18 μm without substrate (**c**) and *t* = 18 μm with Rogers 5880 substrate *t*_*s*_ = 0.787 mm (**d**) corresponding to the red dashed line in (**a**). It is obvious that the field confinements of the proposed PSPW with substrate are much stronger compared with those staggered plasmonic waveguide structures without substrate.

Subwavelength field confinement is one of the key characteristics for the spoof SPP modes, which can be achieved by corrugated plasmonic waveguides. The basic principle to realize tight confinement of spoof SPP for the proposed PSPW is similar to other corrugated single/double-sided bulk or planar plasmonic waveguides. By creating special artificial electromagnetic boundary conditions, PSPWs increase the penetration of the electromagnetic fields into the subwavelength grooves in the metal strip, resulting in tight confinement of spoof SPPs. However, due to their special structural feature with a geometrically lateral shift of *d*/2 between the two groove arrays, the proposed PSPWs exhibit a new waveguiding phenomenon with the advantages of much tighter field confinement. To intuitively view these characteristics, we first compare the spatial distributions of fundamental modes for PSPW and staggered copper plasmonic waveguides (Type II) with the same dimensions at 11.4 GHz, as shown in Fig. 2. The schematic view of electric field vector distribution on the *xoy* plane cut in copper is presented in Fig. 2(a). And Fig. 2(b–d) demonstrate the normalized amplitudes of electric fields (|*E*|) distributions on the cross-section (*yoz* plane) corresponding to the red dashed line in (a) for the Type II waveguide with t → ∞ without substrate, Type II waveguide with *t* = 18 μm without substrate, and PSPW with Rogers 5880 substrate (*t* = 18 μm and *t*_s_ = 0.787 mm). It is obvious that the electric fields at all cases are tightly confined around and rapidly decay away from the corrugated groove area at a deep subwavelength scale. As clearly illustrated in these figures, the proposed PSPW consisting of metallic strip and substrate exhibits much tighter spatial mode confinement and the mode size is reduced over 90% compared with the staggered plasmonic waveguides consisting of only copper with t = 18 μm or ∞ cases, which is consistent with the theoretical prediction from the dispersion relations in Fig. 1(b). Besides the subwavelength field confinement features, this PSPW also has attractive advantages in their planar and miniature structures and great potential in planar plasmonic devices compared with the conventional bulk plasmonic waveguides.

**Figure 3:**
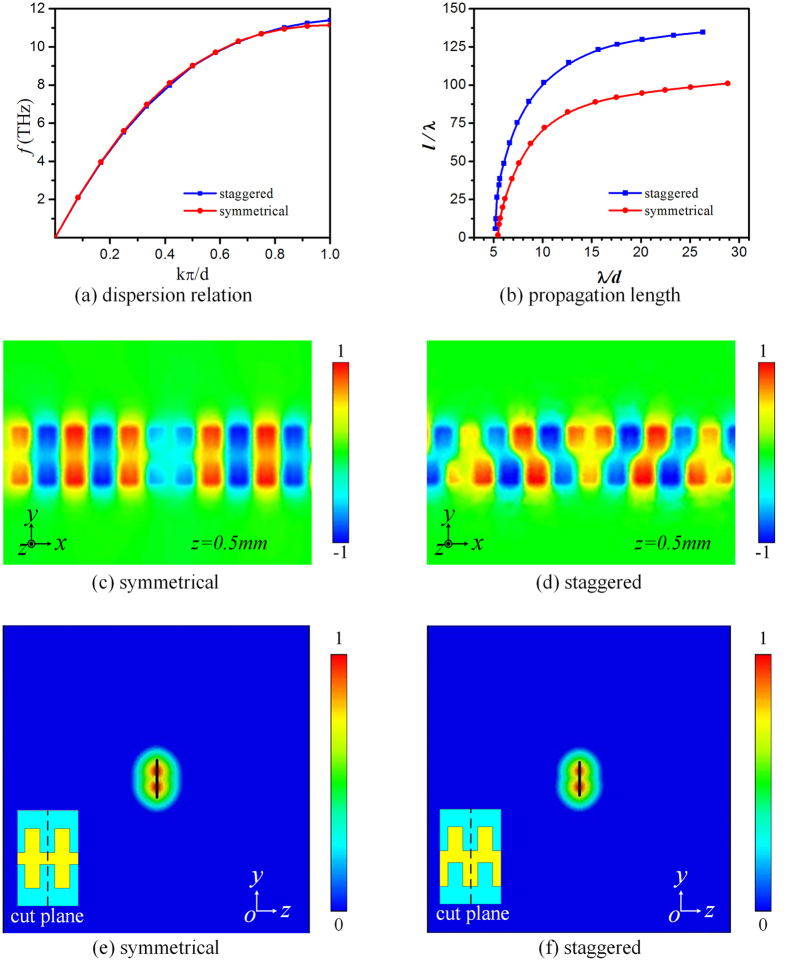
Simulated dispersion relation, propagation length, and near field distributions for the plasmonic waveguides with symmetrical and staggered double-sided corrugation case with the same structural dimensions: (**a**) dispersion relation; (**b**) propagation length; (**c**,**d**) normalized electric field component *E*_*z*_ distributions on the *xoy* plane which is 0.5 mm away from the strip; (**d**,**e**) normalized amplitudes of electric fields (|*E*|) on the *yoz* plane at each asymptotic frequency.

Due to its special structural feature with a geometrically lateral shift, the proposed planar staggered plasmonic waveguide displays unique staggered EM coupling and waveguiding phenomenon. To gain further insight into these propagation properties, we compare the dispersion relation and field distributions of the PSPW with that of the symmetrical double-sided plasmonic waveguide, as shown in Fig. 3. The dispersion relation and propagation length curves calculated from the plasmonic waveguides with symmetrical and staggered double-sided corrugation case with the same structural dimensions are presented in Fig. 3(a,b). Obviously, the dispersion relation curves are close to each other, and the asymptotic frequencies computed at *kπ*/*d* for the staggered structure is 11.4 GHz and the symmetrical structure is 11 GHz, respectively, while, the propagation length for the staggered structure is much longer than the symmetrical structure. Figure 3(c,d) show the electric field *E*_*z*_ profiles for the staggered and symmetrical cases at each asymptotic frequency. It is clear that the EM cross coupling in the *x*-direction of PSPW is much stronger than that of a symmetrical plasmonic waveguide. Figure 3(e,f) display the normalized amplitudes of electric field (|*E*|) distributions on the *yoz* plane for both structures, indicating equivalent tight field confinement for both structures at each asymptotic frequency. Such phenomenon mainly results from the PSPW’s special structural feature with a lateral shift. The distance between the two adjacent grooves in both sides in the PSPW is reduced, and the EM coupling between adjacent units is enhanced, which accordingly results in slightly increasing the asymptotic frequencies and drastically improving the propagation length, while keeping as tight field confinement as the symmetrical double side corrugated plasmonic waveguide. Besides, we also compare the dispersion relation, propagation length and the field distributions among PSPW (with staggered single-strip) and two other kinds of double-strip plasmonic waveguides with U-shaped corrugation (proposed in ref. 35) and staggered corrugation with the same structural dimensions in Supplementary Information. It is found that, though PSPW’s field confinement is very tight, the double-strip structures can demonstrate even stronger subwavelength field confinement effects at the expense of much higher propagation loss and tens of times shorter propagation length. From this point of view, PSPW is a promising spoof SPP waveguide with excellent performance, which not only can achieve subwavelength spoof SPP confinement but also can exhibit low loss and long propagation length.

### CPW-PSPW transition design and experimental verification of the properties of PSPWs

**Figure 4:**
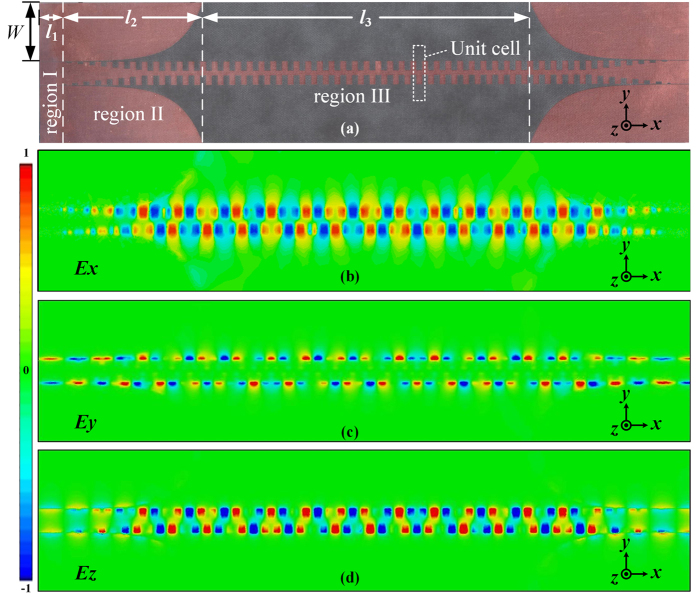
The illustration of CPW-PSPW transitions. (**a**) Fabricated CPW-PSPW-CPW structure sample. (**b**~**d**) Normalized electric field component *E*_*x*_, *E*_*y*_ *and E*_*z*_ distribution at 10.7 GHz on the *xoy* plane which is 0.5 mm away from the copper strip.

Due to the large momentum mismatch between spoof SPPs and the plane wave, it is of great importance to investigate and design smooth transition for highly efficient spoof SPP mode conversion. As we know, the PSPW’s momentum can be manipulated by the groove depth *h* while keeping other parameters fixed (see Fig. S1(a) in Supplementary Information), indicating the possibility of efficient spoof SPP mode conversion using a gradient depth groove structure. Based on this tuning characteristic and borrowing the idea from ref. 32, a broadband CPW to PSPW transition structure at microwave frequencies is designed. Figure 4(a) shows a fabricated CPW-PSPW-CPW structure, where the CPW-PSPW transition is composed of three regions: region I, region II and region ІІІ. In this structure, the thicknesses of copper strip and Rogers RT/duroid 5880 are *t* = 18 μm and *t*_s_ = 0.787 mm, respectively. Region І is the CPW part with characteristic impedance of 50 Ω, *l*_1_ = 10 mm, *g* = 0.4 mm, and *W* = 25 mm, where coaxial to CPW converters will be soldered on during measurement. Region ІІІ is the straight uniform PSPW part with length *l*_2_ = 140 mm (28 periods of unit cells), where *d* = 5 mm, *a* = 2.5 mm, *b* = 2 mm and *h* = 4 mm. And region ІІ is the transition part with flaring ground and gradient depth grooves to achieve smooth momentum and impedance matching for the spoof SPP mode conversion from CPW guided mode. In this region, the flaring ground curve is set with length *l*_3_ = 60 mm, while the center strip includes 8 periods of the gradient depth grooves with the depth linearly varying from 0.5 mm to 4 mm as a step of 0.5 mm and 4 periods of uniform depth grooves consistent with Region III. At the beginning of the transition, the groove depth is small and the flaring ground is needed to confine the guided wave to achieve high transmission efficiency. As the groove depth increases, the quasi-TEM guided wave of CPW in this circuit gradually becomes the spoof SPPs with high confinement, and accordingly, the ground is flared out and terminated. To intuitively and clearly illustrate the smooth mode conversion state between CPW guided waves and spoof SPPs for the CPW-PSPW-CPW structure, we present the simulated normalized near-field distribution of *E*_*x*_ component at 10.7 GHz on the *xoy* plane, which is 0.5 mm away from the copper strip, as shown in Fig. 4(b). In the CPW sections, the electric field vectors of the quasi-TEM guided wave lie mainly on the *yoz* plane and the *E*_*x*_ component is very small, while in region ІI, the quasi-TEM guided wave is gradually transformed to spoof SPPs, so the *E*_*x*_ component increases accordingly. In region III, the fundamental mode of spoof SPPs becomes dominant guided wave propagating along PSPW with little loss. Hence the amplitude of *E*_*x*_ remains almost the same with the phase changing periodically along the x-direction. Similar phenomenon can be observed from the distributions of normalized electric field component *E*_*y*_ and *E*_*z*_ at the same cut plane in Fig. 4(c,d). Therefore, this CPW-PSPW transition has smooth and highly efficient mode conversion performance.

**Figure 5:**
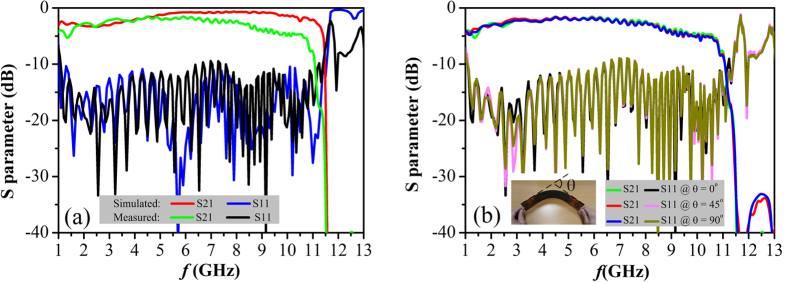
S parameters of the CPW-PSPW-CPWS structure. (**a**) Simulated and measured S parameters. (**b**) The measured S parameters under different bend angles *θ* = 0°, 45°, 90°.

To further experimentally verify the performance of the proposed PSPWs, we measure the fabricated CPW-PSPW-CPW sample shown in Fig. 4(a) using Agilent N5230C PNA microwave network analyzer. For comparison, the simulated and measured S parameters are displayed in Fig. 5(a). It shows that both the simulated and measured S11 parameters are less than −10 dB in the frequency range from 1 GHz to 11.4 GHz, indicating broadband, smooth matching and good mode conversion between PSPW and CPW are realized by this transition structure. Furthermore, both the simulated and measured S21 parameters are almost larger than −5 dB from 1 GHz to 10.8 GHz, and drop below −40 dB when the operating frequency exceeds PSPW’s asymptotic frequency at 11.4 GHz, which agrees well with the numerical prediction of the depression relations in Fig. 1(b). Generally, the experimental results are consistent with the simulated ones, while the slight magnitude difference is mainly caused by introducing two coaxial to CPW converters in the measurement and the fabrication tolerance. Furthermore, in order to investigate the bend loss characteristics of PSPWs, we have measured the S parameters for the whole structure at different bend angles *θ* ranging from 0° to 90°. As is shown in Fig. 5(b), it clearly demonstrates that excellent transmission performance from 1 GHz to 11.4 GHz for bend angles *θ* = 0°, 45° and 90° is achieved with small magnitude difference. Because of the high field confinement characteristics, the microwave signal from the input coaxial probe can be effectively guided to the output probe by this structure. The waveguide performance is insensitive to the bend angle of PSPW structure, and only small additional amount of insertion losses caused even for big bend angle of 90°, showing the PSPW has great conformal waveguide capacity. From the previous analysis, the proposed PSPWs have excellent propagation performance of strong subwavelength spoof SPPs field confinement, long propagation length and low bend loss, great design flexibility, as well as their highly efficient and broadband transition structures, which may have many potential applications in building various planar plasmonic devices and circuits at microwave and terahertz frequencies.

In summary, PSPWs consisting of an ultrathin metallic strip with periodic subwavelength staggered double groove arrays supported by a flexible dielectric substrate are proposed and demonstrated as an efficient type of planar plasmonic waveguides to achieve strong spoof SPPs waveguiding with subwavelength confinement. Unique staggered EM coupling and spoof SPP waveguiding phenomenon have been clearly observed in this PSPW structure. The spoof SPP propagation properties including dispersion relations and near field distributions under different geometric and dielectric parameters are numerically investigated. To verify the PSPW’s propagation performance, smooth and highly efficient broadband CPW-PSPW transitions with flaring ground and gradient depth grooves are designed, and then a CPW-PSPW-CPW structure is fabricated and measured at microwave frequencies. Both numerical simulated and measured results validate the efficient waveguiding of spoof SPPs in a wide frequency range from 1 GHz to 11.4 GHz. The investigation results show the proposed PSPWs have promising performance of strong subwavelength spoof SPPs field confinement, long propagation length and low bend loss, as well as great flexibility to engineer the propagation properties by adjusting their geometry dimensions and material parameters, which may have many potential applications in integrated plasmonic devices and circuits in microwave and terahertz regimes.

## Methods

In the numerical part, we use the finite element method implemented in commercial software ANSYS HFSS to numerically calculate the properties of the Spoof SPPs propagating along the PSPWs in *x*-direction. The dispersion curves are obtained by calculating the eigen frequencies of the unit cell structure (see Fig. 1) with different phase shifts between the Master and Slave boundaries in *x*-direction using Eigenmode solution. The boundaries in *y* and *z* directions are set as “PML” at large distances from the waveguide structure to simulate the real space and avoid spurious reflections. Then, the near field electric field distributions are plotted at the asymptote frequency. While for the CPW-PSPW-CPW structure, Driven Modal is used to calculate the S parameters and electric field distributions. The detailed parameters of the unit structure are illustrated in the main text.

In the experimental part, the CPW-PSPW-CPW structure samples are fabricated using a standard etching process on 0.787 mm thickness Rogers RT/duroid 5880 substrate, whose dielectric constant *ε*_r_ = 2.2 and loss tangent tan(δ) = 0.0001, and copper layer’s thickness is 18 μm. Then, SMA connectors are soldered on the CPW parts of these samples. In the measurement, Agilent N5230C PNA microwave network analyzer is used to feed guided waves and measure the S parameters for the CPW-PSPW-CPW structures at different bend angles *θ* ranging from 0° to 90°.

## Additional Information

**How to cite this article**: Ye, L. *et al*. Strongly Confined Spoof Surface Plasmon Polaritons Waveguiding Enabled by Planar Staggered Plasmonic Waveguides. *Sci. Rep.* **6**, 38528; doi: 10.1038/srep38528 (2016).

**Publisher's note:** Springer Nature remains neutral with regard to jurisdictional claims in published maps and institutional affiliations.

## Supplementary Material

Supplementary Information
