# Supplementary material for: Strongly Confined Spoof Surface Plasmon Polaritons Waveguiding Enabled by Planar Staggered Plasmonic Waveguides
Source: Sci Rep. 2016 Dec 5;6:38528. doi: 10.1038/srep38528 (PMC5137036; doi:10.1038/srep38528)
Supplement: Supplementary Information [file srep38528-s1.pdf]

Supplementary Information

Strongly Confined Spoof Surface Plasmon Polaritons Waveguiding Enabled by Planar Staggered Plasmonic Waveguides

Longfang Ye[1], Yifan Xiao[1], Yanhui Liu[1], Liang Zhang[1], Guoxiong Cai[1], and Qing Huo Liu[2]*

[1]Institute of Electromagnetics and Acoustics, and Department of Electronic Science, Xiamen University, Xiamen 361005, China

[2]Department of Electrical and Computer Engineering, Duke University, Durham 27708, USA

*qhliu@duke.edu

1. Dependence of the dispersion relations on the geometric and dielectric parameters of PSPWs

Fig. S1 shows the dependence of asymptotic frequency on the groove depth h and the period d. As shown in Fig. S1(a), the dispersion curves for fundamental spoof SPP modes are calculated for several different values of the depth h, ranging from h = 2 mm to 6 mm while keeping d = 5 mm, a = 2.5 mm, b = 2 mm, t = 18 μm and t_s = 0.787 mm fixed. The asymptotic frequency decreases dramatically from 17.6 GHz to 8.7 GHz as h increases from 2mm to 6 mm (see the inset curve). Similarly, by varying the period d while fixing h = 4 mm and the ratio of a / d = 0.5, the variation of the dispersion relations for fundamental spoof SPP modes is presented in Fig. S1(b). It is found that the deeper the groove or the larger the period is, the lower asymptotic frequency and the stronger field confinement become.

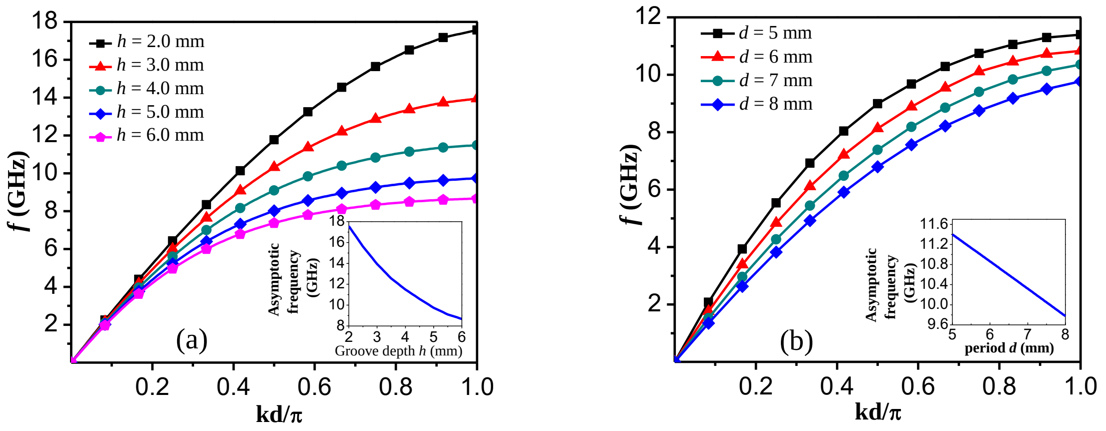

Fig. S1 Variation of the dispersion relations for the fundamental spoof SPPs as a function of geometric parameters: (a) the groove depth *h*, with *d* = 5 mm, *a* = 2.5 mm, *t* = 18 μm and t_s = 0.787 mm fixed for all curves, (b) the groove period *d*, with *a* = 0.5 *d*, *h* = 4 mm, *t* = 18 μm and t_s = 0.787 mm fixed for all curves.

Fig. S2(a) and (b) indicate the dispersion curves as a function of relative permittivity ε_r and thickness t_s, respectively, while keeping *d* = 5 mm, *a* = 2.5 mm, *b* = 2 mm, *t* = 18 μm and *h* = 4 mm fixed. As illustrated in this Fig. S2(a), as the dispersion curve deviates farther away from the light line, lower asymptotic frequency and stronger confinement can be achieved as relative permittivity ε_r increases. And the asymptotic frequency drops rapidly from 11.7 GHz to 7.6 GHz as the ε_r increases from 2 to 10, as shown in the inset curve. Thus, we can manipulate the spoof SPPs' operating frequencies and field confinement by selecting different substrates for PSPW to satisfy various demands with fixed geometry. Fig. S2(b) displays the dependence of the dispersion relations on the thickness of substrate. By selecting Rogers 5880 as substrate with ε_r = 2.2 and fixing other geometric parameters, the dispersion curves under five values of the thickness t_s ranging from 0.1 mm to 3 mm is calculted. It is obvious that the asymptotic frequency decreases as the t_s increases. And this decreasing trend is relatively faster with t_s smaller than 2 mm, while it becomes less sensitive to t_s variation when t_s exceeds 2 mm, which is mainly resulting from the extremely subwavelength field confinement near

the copper strip.

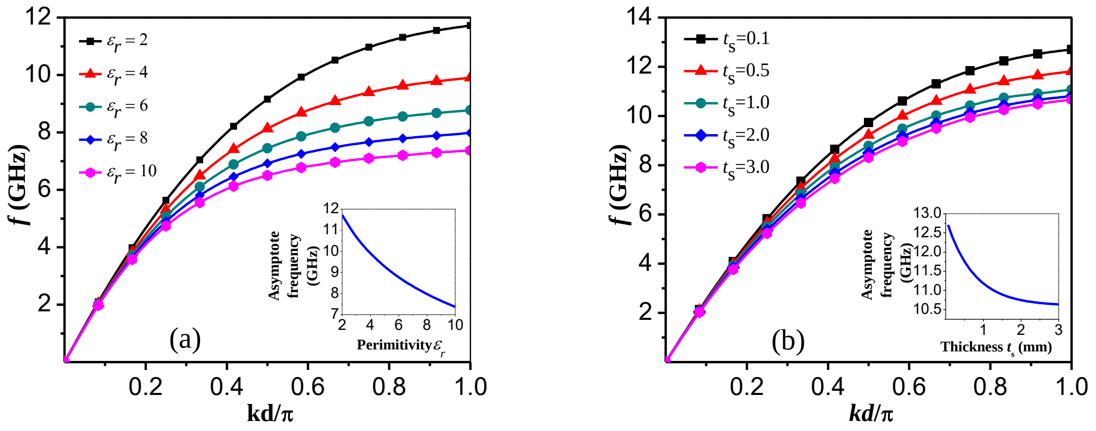

Fig. S2 Variation of the dispersion relations for the fundamental spoof SPPs as a function of the substrate relative permittivity ε_r (a) and thickness t_s (b), respectively, while keeping d = 5 mm, a = 2.5 mm, t = 18 μm and h = 4 mm fixed.

The numerical results show that the operating frequency band of PSPW can be extended to terahertz or infrared regimes by proportionally reducing the geometric parameters of the PSPW's unit cell. Therefore, by combining such geometric and dielectric parameters tuning, the PSPWs demonstrate huge design flexibility to engineer the spoof SPP dispersion relations with great potential applications in various planar plasmonic devices and circuits at different frequencies.

2. Comparison among single-strip PSPW and double-strip plasmonic waveguides with U-shaped corrugation and staggered corrugation

Fig. S3 presents a comparison of dispersion relation, propagation length and the field distributions among single-strip PSPW and double-strip plasmonic waveguides with U-shaped corrugation and staggered corrugation under same structural dimensions. Double-strip plasmonic waveguide with U-shaped corrugation, proposed in Ref. 35 (main text), is composed of two corrugated metallic strips that are printed antisymmetrically on the top and bottom surfaces of a dielectric substrate. Similarly, double-strip plasmonic waveguide is consist of two corrugated metallic strip staggered corrugation printed antisymmetrically on

a dielectric substrate. As shown in Fig. S3(a), we found that the asymptotic frequency of the U-shaped double-strip plasmonic waveguide is much lower than that of the PSPW (single-strip). Though PSPW's field confinement is very tight, the U-shaped double-strip structure demonstrates even stronger subwavelength field confinement effects, as shown in Fig. S3(c) and (d). That is mainly attribute to the strong EM coupling between such two corrugated metallic strips in that double-strip waveguide, resulting in large enhancement of its equivalent capacitance and inductance. Hence, it is able to decrease the plasma frequency and achieve stronger field confinement effects. Following this scheme, similar dispersion relation and field confinement characteristics can be obtained in double-strip plasmonic waveguides with staggered corrugation, as shown in Fig. S3(a) and (e). However, such double-strip structures may suffer from the disadvantages of much higher propagation loss and much shorter propagation length. As clearly observed in Fig. S3(b), the propagation length of PSPW (single-strip) is 6 times to tens of times longer than the double-strip structures. Obviously, there is a trade-off between propagation length and field confinement depending on different practical applications. From this point of view, PSPW is a promising spoof SPP waveguide, which has excellent performance with tight confinement and low propagation loss. In this paper, we mainly focus on the single-strip PSPW, which not only can exhibit low loss and long propagation length, but also can achieve sufficient subwavelength spoof SPPs confinement enabling low bend loss even at large bend angle of 90°.

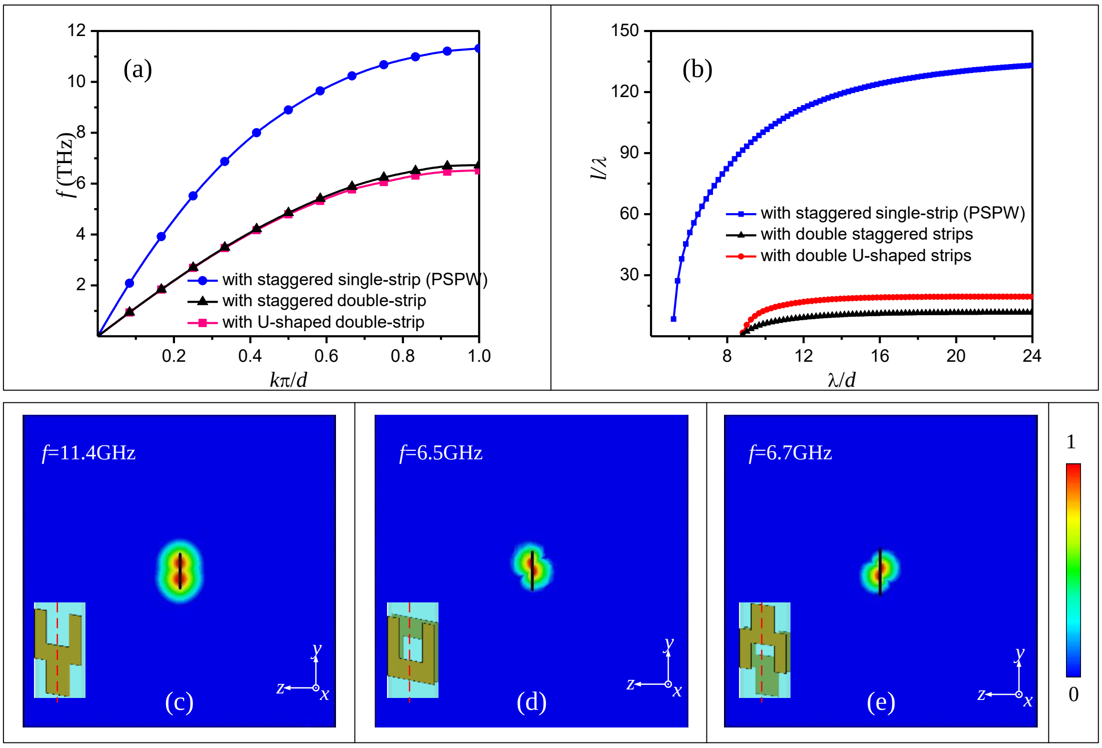

Fig. S3 Simulated dispersion relation, propagation length and near field distributions for different kinds of plasmonic waveguides with staggered single-strip, U-shaped double-strip and staggered double-strip with same structural dimensions: (a) dispersion relation, (b) propagation length, (c)~(e) normalized electric field (|E|) distributions at each asymptotic frequency.
